# Reconstruction of cheek mucosal defect with a buccal fat pad flap in a squamous cell carcinoma patient: a case report and literature review

**DOI:** 10.1186/s40902-018-0150-8

**Published:** 2018-05-25

**Authors:** Dae-Seok Hwang, Jinyoung Park, Uk-Kyu Kim, Hae-Ryoun Park, Gyoo-Cheon Kim, Mi-Heon Ryu

**Affiliations:** 1 Department of Oral and Maxillofacial Surgery, School of Dentistry, Pusan National University Dental Hospital, 20, Geumo-ro, Mulgeum-eup, Yangsan, Gyeongsangnam-do South Korea; 2 Institute of Translational Dental Sciences, Pusan National University, Yangsan, South Korea; 3 Dental Research Institute, Pusan National University Dental Hospital, Yangsan, South Korea

**Keywords:** Buccal fat pad flap, Buccal mucosal defect, Buccal fat pad, Oral cavity reconstruction, Pedicled buccal fat pad flap

## Abstract

**Background:**

Squamous cell carcinoma (SCC) is the most commonly occurring malignant tumor in the oral cavity. In South Korea, it occurs most frequently in the mandible, tongue, maxilla, buccal mucosa, other areas of the oral cavity, and lips. Radial forearm free flap (RFFF) is the most widely used reconstruction method for the buccal mucosal defect. The scar of the forearm donor, however, is highly visible and unsightly, and a secondary surgical site is needed when such technique is applied. For these reasons, buccal fat pad (BFP) flap has been commonly used for closing post-surgical excision sites since the recent decades because of its reliability, ease of harvest, and low complication rate.

**Case presentation:**

In the case reported herein, BFP flap was used to reconstruct a cheek mucosal defect after excision. The defect was completely covered by the BFP flap, without any complications.

**Conclusion:**

Discussed herein is the usefulness of BFP flap for the repair of the cheek mucosal defect. Also, further studies are needed to determine the possibility of using BFP flap when the defect is deep, and the maximum volume that can be harvested considering the changes in volume with age.

## Background

Squamous cell carcinoma (SCC) accounts for about 90% of the malignant tumors occurring in the oral cavity [1, 2]. SCC can occur in any part of the mouth, but in South Korea, it occurs most frequently in the mandible, tongue, maxilla, buccal mucosa, other areas of the oral cavity, and lips. The incidence of oral cancer in the mandible and buccal mucosa has been increasing since 2001 [3].

The treatment of SCC in the buccal mucosa consists of a wide surgical excision, and it is essential to achieve negative margins [4]. After a wide surgical excision, the buccal mucosal defect is most commonly reconstructed through radial forearm free flap (RFFF). This technique, however, leaves a scar on the forearm donor that is highly visible and unsightly. Moreover, it requires a secondary surgical site [5–9]. For these reasons, buccal fat pad (BFP) flap has been commonly used for closing surgical excision sites since the recent decades because of its reliability, ease of harvest, and low complication rate [10–17]. A buccal mucosa defect can be successfully covered with a pedicled BFP flap, which was first described by Egyedi in 1977 for the closure of oroantral and oronasal communications secondary to oncologic resections [10].

Since then, many studies have reported the anatomy of BFP with its blood supply and the large number of cases with a small number of complications [11–22]. Presented herein is a patient diagnosed with SCC on the left-cheek mucosa. The malignant tumor was excised, and the defect was covered with a simple pedicled BFP flap.

## Case presentation

**Fig. 1:**
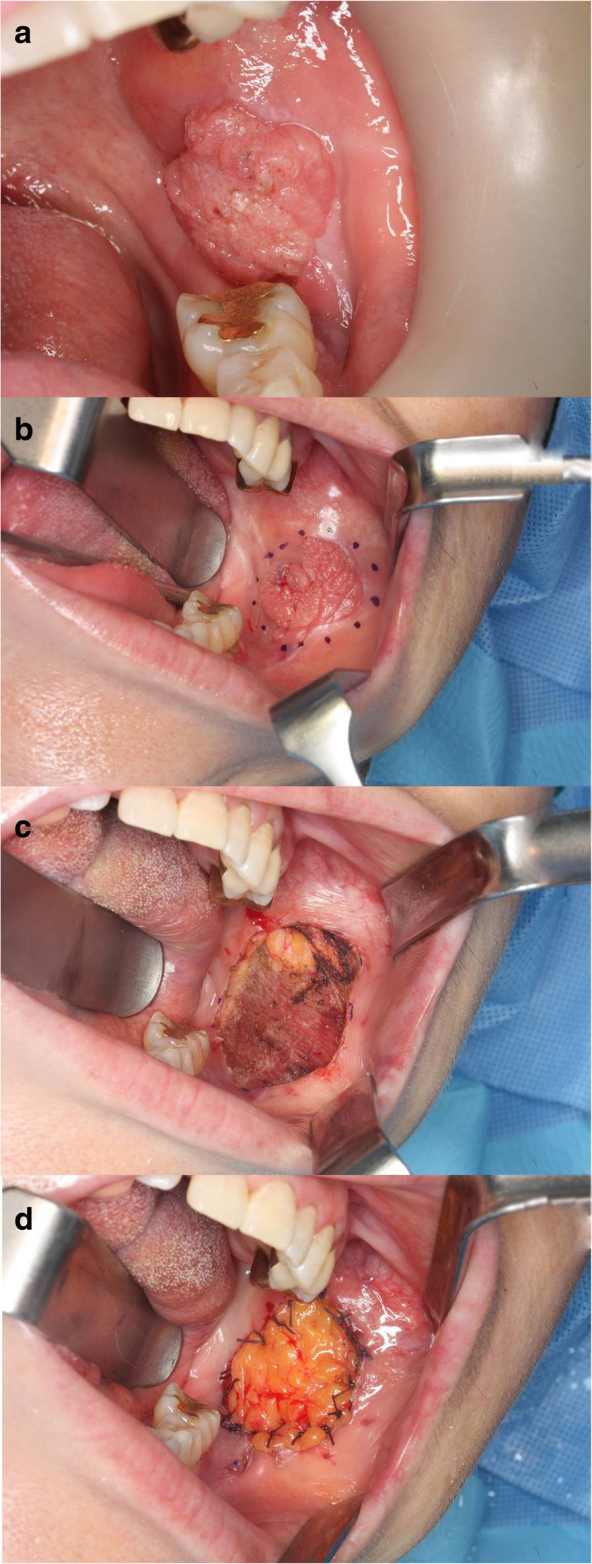
**a** Clinical photograph on first visit. **b** Preoperative lesion. **c** Lesion was excised. **d** The immediate post-operative result

**Fig. 2:**
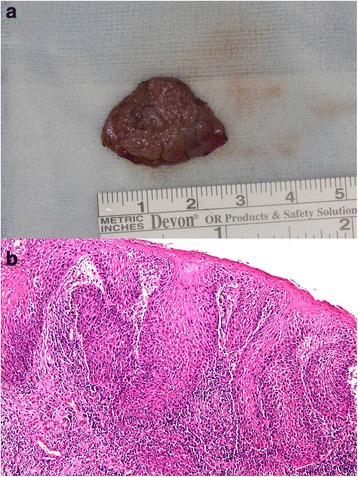
**a** Gross anatomy. Macroscopically, the excised specimen had dimensions of 2.1 × 2.0 × 0.3 cm. **b** Microscopic examination (H&E stain) confirmed well-differentiated SCC

In October 2015, a 55-year-old female patient was referred to the author’s hospital because of the recurrence of a lesion in the left buccal mucosa. The patient was diagnosed with squamous epithelial hyperplasia after excisional biopsy and histological examination in February 2015, and recurrence was confirmed during the follow-up in September 2015. Upon the initial examination at Pusan National University Dental Hospital, a 2-cm exophytic lesion was observed in the left buccal mucosa (Fig. 1a), and the patient did not have any symptom. Also, no invasion of the left mandible was observed on the panoramic view. Excision and reconstruction by BFP flap was planned under general anesthesia considering the clinical and radiological findings obtained on November 11, 2015. The size of the removed lesion with a safety margin was 2.1 × 2.0 × 0.9 cm (Fig. 2a), and the buccal mucosa defect was reconstructed as a pedicled BFP flap (Fig. 1b–d). The buccal mucosal defect was successfully covered without any tension. The specimen was examined via H&E staining and immunostaining for Ki-67 and p53. The histological examination showed that the atrophic epithelium was composed of dysplastic squamous cells with no maturation pattern. Moreover, the epithelial cells displayed active invasion of the underlying stromal tissue (Fig. 2b). Upon immunohistochemical examination, both Ki-67 and p53 were found to be positive. Therefore, the lesion was diagnosed as a well-differentiated SCC.

**Fig. 3:**
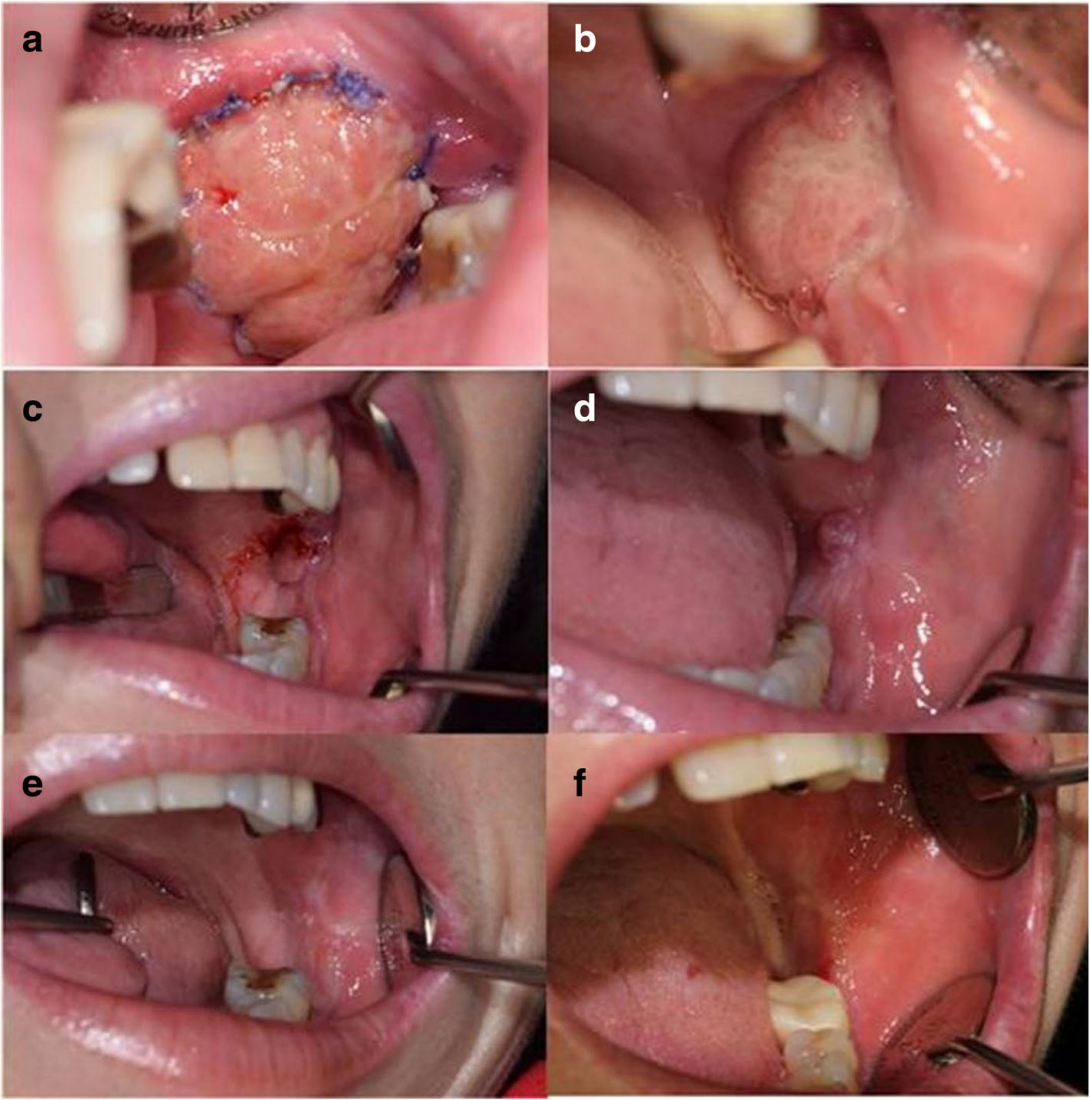
Re-epithelialization of the grafted buccal fat pad after operation. **a**. The ninth day. **b** After 3 weeks. **c** After 1 month. **d** After 2 months. **e** After 3 months. **f** After 1 year

After the diagnosis of malignancy, the patient was subjected to magnetic resonance imaging (MRI), computed tomography (CT), and positron emission tomography with 2-deoxy-2-fluorine-18-fluoro-D-glucose integrated with computed tomography (18-FDG PET/CT) to determine the treatment direction and cancer stage (Fig. 3). It was found from the 18-FDG PET/CT, MRI, and CT that there was no remnant tumor or no significant cervical lymphadenopathy.

**Fig. 4:**
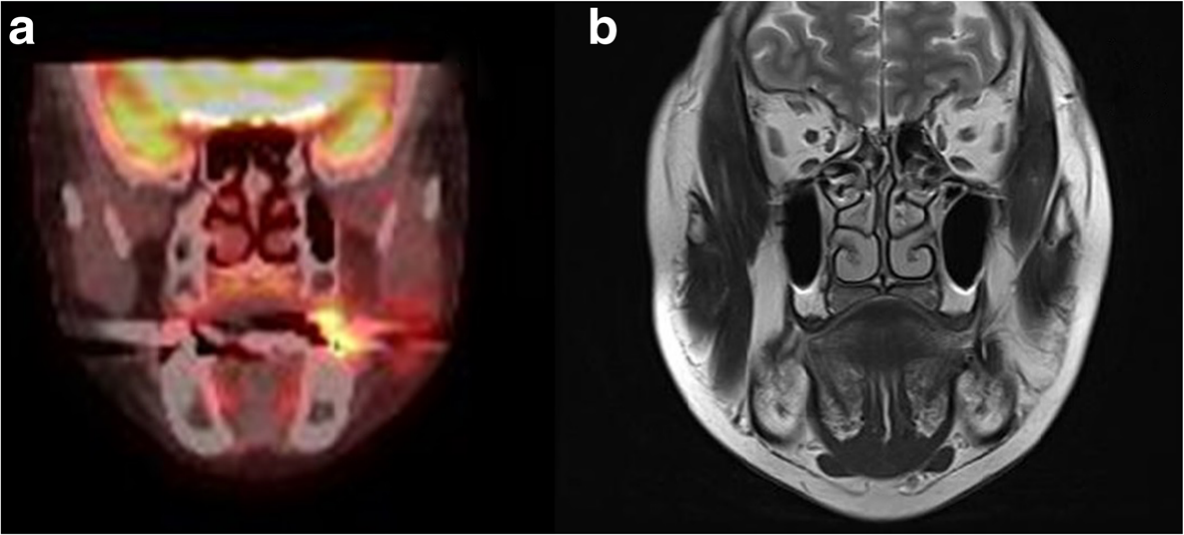
Radiological findings showing post-op state change on left buccal area. **a** 18-FDG PET/CT coronal view showing hot FDG spot on left buccal area because of post-operative change. **b** T2-weighted magnetic resonance image, coronal view

Epithelialization of the grafted BFP was observed on 9 days, 3 weeks, 1 month, 2 months, 3 months, and 1 year after surgery (Fig. 4). The defect was completely covered, without any complications. There was no evidence of recurrence or distant metastasis at every follow-up visit until 2 years after the surgery.

## Discussion

In the case reported herein, a buccal mucosal defect was reconstructed with BFP flap. The oral defect was covered by the BFP in the posterior maxilla, hard and soft palate, and retromolar region after teeth extraction, saucerization, and tumor excision [14, 15, 17].

BFP is an encapsulated mass of adipose tissue in the oromaxillofacial region located in the buccal space between the buccinator muscle and the mandibular ramus and masseter muscle. The BFP has four extensions of the central body: the buccal, pterygoid, pterygopalatine, and temporal extensions [11, 14, 17]. The central body and buccal extension account for approximately 50% of the BFP and are the most clinically significant parts. These are most commonly transposed to cover oral defects [13].

BFP has three sources of blood supply: the maxillary artery (buccal and deep temporal branches), superficial temporal artery (transverse facial branch), and facial artery (small branches). Due to its rich blood supply, the use of BFP flap has a high success rate [12, 15].

Many authors have introduced BFP flap as the safest reconstructive method for small to medium-sized intraoral defects. Martin-Granizo et al. [17] reported that compared to RFFF, the most notable advantages of BFP flap are that it requires a simple and rapid surgical technique, has a low complication rate, and has predictable results without any esthetic sequela. Furthermore, the risk of infection is reduced because BFP is located in the same surgical area as the defect to be covered. Tideman et al. [11] reported the ability to close defects up to 60 × 50 × 30 mm in size using BFP. Moreover, it covers the areas from the premolar area to the posterior tuberosity in the maxilla, retromolar trigone, buccal mucosa, and anterior tonsillar pillar. It must be sutured to the margins of the defect without tension, however, to prevent necrosis of the flap [15].

Pedicled BFP flap, however, has several complications. Past studies described cheek depression after transferring a large volume of buccal fat [13, 14]. Also, the unexpected complication of mouth opening limitation reported by the previous literatures may result from the dense fibrous connective tissue in the subepithelial stroma lacking lamina propria and submucosa [23].

To minimize the incidence of postoperative complications, it is suggested that the patient receive a liquid or soft, non-chewy diet until BPF epithelialization [15]. Epithelialization takes place within 4–6 weeks. Loukas et al. [24] found that the mean volume of BPF is 10.2 ml in males and 8.9 ml in females, with a 6 mm thickness and a 9.7 g mean weight. Based on a review of the literature, the use of BFP has increased due to its advantages. Few studies, however, have investigated the volumetric variations in BPF among age and gender groups.

Before the use of pedicled BFP flap for the reconstruction of defects, the individual volume of BFP needs to be calculated from radiographic images such as CT or MRI images to assess if coverage is possible. Further studies are needed to determine the possibility of using BFP flap when the defect is deep, and the maximum volume that can be harvested considering the changes in volume with age.

## Conclusion

When reconstructing a buccal defect in the oral cavity, pedicled buccal fat pad (BFP) flap is useful. If the defect is small, as in the patients described herein, reconstruction with BFP flap may produce good results. More studies are needed, however, to determine the maximum volume that can be harvested and the size that can be covered by BFP flap considering gender, age, and individual variations.

## Abbreviations

18-FDG PET/CT: Positron emission tomography with 2-deoxy-2-fluorine-18-fluoro- D-glucose integrated with computed tomography
BFP: Buccal fat pad
CT: Computed tomography
MRI: Magnetic resonance imaging
RFFF: Radial forearm free flap
SCC: Squamous cell carcinoma
